# Spontaneous pulmonary hypertension in genetic mouse models of natural killer cell deficiency

**DOI:** 10.1152/ajplung.00477.2017

**Published:** 2018-09-20

**Authors:** Matthew T. Rätsep, Stephen D. Moore, Salema Jafri, Melissa Mitchell, Hugh J. M. Brady, Ofer Mandelboim, Mark Southwood, Nicholas W. Morrell, Francesco Colucci, Mark L. Ormiston

**Affiliations:** ^1^Departments of Biomedical and Molecular Sciences, Medicine, and Surgery, Queen’s University Kingston, Ontario, Canada; ^2^Department of Medicine, University of Cambridge, Cambridge, United Kingdom; ^3^Imperial College, London, United Kingdom; ^4^Hebrew University of Jerusalem, Jerusalem, Israel; ^5^Department of Obstetrics and Gynecology, University of Cambridge, Cambridge, United Kingdom

**Keywords:** angiopoietin-2, interleukin-23, natural killer cells, pulmonary hypertension, Th17

## Abstract

Natural killer (NK) cells are cytotoxic innate lymphoid cells with an established role in the regulation of vascular structure in pregnancy and cancer. Impaired NK cell function has been identified in patients with pulmonary arterial hypertension (PAH), a disease of obstructive vascular remodeling in the lungs, as well as in multiple rodent models of disease. However, the precise contribution of NK cell impairment to the initiation and progression of PAH remains unknown. Here, we report the development of spontaneous pulmonary hypertension in two independent genetic models of NK cell dysfunction, including *Nfil3^−/−^* mice, which are deficient in NK cells due to the absence of the NFIL3 transcription factor, and *Ncr1-Gfp* mice, which lack the NK activating receptor NKp46. Mouse models of NK insufficiency exhibited increased right ventricular systolic pressure and muscularization of the pulmonary arteries in the absence of elevated left ventricular end-diastolic pressure, indicating that the development of pulmonary hypertension was not secondary to left heart dysfunction. In cases of severe NK cell impairment or loss, a subset of mice failed to develop pulmonary hypertension and instead exhibited reduced systemic blood pressure, demonstrating an extension of vascular abnormalities beyond the pulmonary circulation into the systemic vasculature. In both mouse models, the development of PAH was linked to elevated interleukin-23 production, whereas systemic hypotension in *Ncr1-Gfp* mice was accompanied by a loss of angiopoietin-2. Together, these results support an important role for NK cells in the regulation of pulmonary and systemic vascular function and the pathogenesis of PAH.

## INTRODUCTION

Natural killer (NK) cells are innate lymphoid cells (ILCs) that are traditionally charged with the identification and lysis of stressed, oncogenically transformed, or virally infected cells. Beyond this role as the cytotoxic effectors of innate immunity, multiple reports have also highlighted a role for NK cells in the regulation of vascular structure, integrity, and tone (6, 19, 42, 48). This vascular activity has been particularly well documented in pregnancy, where NK cells in the decidual lining of the pregnant uterus are believed to influence the expansion of the spiral arteries that supply oxygen and nutrients to the developing fetus (3, 19, 26). Although this functionality was originally believed to be limited to the specialized NK cells of the uterus, recent studies have demonstrated a potential role for NK cells in the regulation of other vascular beds (6, 23, 48), including the pulmonary circulation (31, 49).

Pulmonary arterial hypertension (PAH) is a disease of occlusive vascular remodeling that is marked by the cancer-like (35) proliferation of pulmonary vascular endothelial and smooth muscle cells at the level of the precapillary lung arterioles. The idiopathic and familial forms of this vascular pathology are often accompanied by immune dysfunction (38), including increased inflammatory cytokines (44), autoantibodies (39), ectopic pulmonary lymphoid hyperplasia (36), and elevated transforming growth factor-β (TGFβ) signaling (5). The prevalence of PAH is also substantially elevated in cases of viral infection, such as human immunodeficiency virus, as well as in association with certain autoimmune disorders, including scleroderma and lupus (38). While the precise link between altered immunity and pathological vascular remodeling has yet to be determined, our previous work has identified an impairment of NK cells isolated from patients with PAH, defined by reduced cytolytic function, increased TGFβ signaling, and diminished expression of multiple surface receptors, including KIR3DL1, NKp46, and CD56 (31). NK cells from patients with PAH also exhibit an elevated production of matrix metalloproteinase-9, implicating a capacity for these cells to directly influence pathological vascular remodeling. Supporting data from standard rat and mouse models of PAH also show an overall reduction of NK cell number and function (31).

Although previous studies have demonstrated a role for NK cell stimulation in the mode of action of experimental pharmacological and cell-based PAH therapies (32, 33), it has not yet been determined if NK cell impairment is a driving factor in the initiation and progression of PAH or simply a consequence of established disease.

In the current study, we address this question through the assessment of cardiopulmonary function in two independent genetic mouse models of NK cell insufficiency, the NK-deficient *Nfil3* knockout mouse (15) and the *Ncr1-Gfp* mouse, in which *Gfp* has been inserted into the endogenous locus of gene-encoding NK-activating receptor, NKp46 (16). Mice lacking the NFIL3/E4BP4 basic leucine zipper transcription factor (*Nfil3^−/−^*) are severely deficient in circulating NK cells, as well as ILC subsets (15, 17, 41), but retain NFIL3-independent tissue resident NK (trNK) cells, which are found in a variety of organs, including the uterus, liver, and skin (43). Recent studies examining the contribution of uterine NK cells to vascular remodeling in pregnancy have shown that, despite the persistence of residual trNK cells in the uteruses of *Nfil3^−/−^* mice, these animals exhibit impaired expansion of the uterine arteries during gestation, accompanied by fetal growth restriction when compared with NK-replete wild-type (WT) controls (7).

Unlike *Nfil3^−/−^* mice, *Ncr1-Gfp* animals are not grossly deficient in specific NK or ILC subsets. However, the loss of NKp46 holds the potential to impair the function of all NKp46^+^ cell types, including conventional NK cells, NKp46^+^ ILC populations, and trNK cells that are not impacted by the *Nfil3^−/−^* model. Previous work has shown that C57Bl/6 mice homozygous for the green fluorescent protein (GFP) knock-in (*Ncr1^gfp/gfp^*) exhibit reduced NK cell function, as exemplified by enhanced susceptibility to influenza virus infection (16) and impaired killing of certain lymphoma cell lines (18). *Ncr1-Gfp* mice are also particularly relevant to PAH, as NKp46 expression is reduced in NK cells isolated from humans with the disease (31).

We report the development of spontaneous pulmonary hypertension (PH) in both the *Nfil3^−/−^* and *Ncr1-Gfp* models of NK cell insufficiency, as exemplified by increased right ventricular systolic pressure (RVSP) and muscularization of the pulmonary arteries. Importantly, this elevation of RVSP was observed in the absence of elevated left ventricular end-diastolic pressure (LVEDP), indicating that disease in these mice was not secondary to left heart failure. In both models, a subset of mice failed to develop PH and instead exhibited reduced systolic blood pressure, indicating that, in cases of severe NK cell impairment or loss, vascular abnormalities can extend beyond the pulmonary circulation and impact systemic vascular function. These findings strongly support a role for NK cells in the maintenance of pulmonary and systemic vascular homeostasis and suggest that NK cell impairment is an important contributor to the pathogenesis of PAH.

## MATERIALS AND METHODS

### Mice

*Nfil3^−/−^* and *Ncr1-GFP* mice were generated as described previously (15, 16). All mice were housed in individually ventilated cages and given sterilized food and water. Breeding for the *Nfil3^−/−^* and *Ncr1-GFP* strains involved the mating of sires and dams that were heterozygous for the modified allele, resulting in the production of WT offspring that were used as controls in all studies. All studies were performed in a manner that was blinded to mouse genotype. Mice were identified by animal numbers, with genotypes assigned following the completion of all data acquisition and analysis. All animal studies were conducted under Ethics Board-approved protocols in accordance with the guidelines of the Canadian Council on Animal Care and the United Kingdom Animals (Scientific Procedures) Act 1986 under the approval of the United Kingdom Home Office.

### Preparation of Single Cell Suspensions

At 8 wk of age, mice were bled into tubes coated with EDTA. Fresh blood was incubated in red blood cell (RBC) lysis buffer (BioLegend) for 4 min at room temperature before staining for flow cytometry as described below. To collect lung and spleen, mice were anesthetized with sodium pentobarbital (200 mg/kg ip) and euthanized by exsanguination. Whole spleens were dissected free of any connective tissue and collected into 5 ml ice-cold PBS. The whole spleen was then homogenized using the plunger of a sterile syringe before filtering through a 40-μm nylon mesh strainer (Fisher). The resultant cell suspension was then pelleted at 300 *g* for 5 min and resuspended in 1 ml RBC lysis buffer (BioLegend) for 3 min at room temperature. Cells were washed in PBS and then proceeded to staining for flow cytometry.

To collect lungs, the pulmonary vasculature was perfused with 5 ml PBS containing 20 U/ml heparin through the right ventricle. The entire left lobe was excised, minced with 2 razor blades, and incubated in digestion buffer (RPMI containing 2% FBS, 0.1 U/ml liberase, and 30 μg/ml DNase) for 30 min at 37°C. Digested lung suspensions were then filtered through a 40-μm nylon mesh strainer, washed with PBS, and lymphocytes separated on a Percoll gradient. The resultant cell suspension was then washed with PBS and incubated in 1 ml RBC lysis buffer for 3 min at room temperature before proceeding to staining for flow cytometry.

### Flow Cytometry

Fluorescently conjugated antibodies directed toward mouse antigens CD45, CD3, CD11b, CD27, NK1.1, NKp46, and DX5 were purchased from BioLegend. Rat anti-mouse EOMES was purchased from eBioscience. Hamster anti-mouse CD49a was purchased from BD Biosciences. Surface antigens were stained in PBS containing 0.5% BSA and 1 mM EDTA. For intracellular staining of EOMES, cells were fixed and permeabilized using the Foxp3/Transcription Factor Staining Buffer Set (eBioscience). Cell viability was assessed using 7-aminoactinomycin D (Tonbo Bioscience) in blood or Zombie-NIR Fixable Viability dye (BioLegend) in spleen and lung cell suspensions.

### Cardiopulmonary Phenotyping

#### Echocardiography.

Transthoracic echocardiography was performed using a Vevo 770 instrument (VisualSonics) equipped with a 707B imaging transducer set at 30 MHz. Echocardiography was performed on *Nfil3* and *Ncr1-Gfp* mice at 9 and 4 mo of age, respectively. Mice were anaesthetized with isoflurane (induced at ~3% and maintained at 2%) and placed on a heated table. Electrodes monitored heart and respiration rate, and a rectal thermometer measured temperature. Heart rate was maintained at ~500 beats/min with a mean respiratory rate of ~100 breaths/min. A parasternal short axis view of the left ventricle was obtained at the level of the papillary muscles. An M-mode sample was placed to the center of the left ventricle and across the anterior and posterior pericardium to measure end-systolic LV inner diameter (LVIDs) and end-diastolic LVID diastolic (LVIDd). LV volume diastole (LV Vol;d) was calculated as [7.0/(2.4 + LVID;d)] × LVID;d^3^. LV volume systole (LV Vol;s) was calculated as [7.0/(2.4 + LVID;d)] × LVID;s^3^. LV ejection fraction (EF) was estimated as [LV Vol;d − (LV Vol;s/LV Vol;d)] × 100.

#### Cardiac catheterization.

Closed-chest cardiac catheterization was performed using an MPVS Ultra Single Segment Pressure-Volume Unit in combination with an SPR-839 pressure-volume catheter (both Millar Instruments, Houston, TX). Catherization was performed on mice immediately following echocardiography imaging. Pressure and volume measurements were acquired and analyzed using LabChart Pro software (ADInstruments, Sydney, Australia). Mice were anaesthetized with isoflurane (induced at ~3% and maintained at 2%), and measurements from the right ventricle were collected by catheterization of the right external jugular vein. The catheter was then removed, and the jugular was tied off. Measurements from the left ventricle of the same mice were then collected via catheterization of the right carotid artery. Measurements of systemic blood pressure were collected from the aortic arch before entry into the left ventricle. All measurements were collected at a mean heart rate of ~400–450 beats/min.

Mice were euthanized immediately following catheterization, and the hearts and lungs were harvested. RV hypertrophy was determined as a ratio of RV to LV and septal weight (RV/LV + S). The right lung was snap-frozen in liquid nitrogen for protein and gene expression analysis. The left lung was inflated with a 1:1 mixture of saline and optimal cutting temperature medium (Sakura, Zoeterwoude, The Netherlands) and fixed with 4% paraformaldehyde in PBS before dehydration and paraffin embedding.

### Histology

Assessment of pulmonary arteriolar muscularization was performed as described previously (27). Briefly, sections of fixed mouse lung tissue (5 μm) were labeled with monoclonal mouse anti-mouse/rat/human smooth muscle a-actin (clone 1A4, Dako, Glostrup, Denmark) following preconjugation with a biotinylated anti-mouse immunoglobulin using the Dako ARK system. In accordance with the manufacturer’s instructions, the biotin-labeled primary antibody was detected with streptavidin-peroxidase, followed by diaminobenzidine/hydrogen peroxide as substrate-chromogen. A minimum of 20 alveolar duct-associated arterioles were categorized as either fully, partially, or nonmuscularized. Statistical significance was assessed by comparing the percentage of nonmuscularized vessels between groups. Assessment of lung perivascular cuffing was performed following elastic van Gieson staining of fixed lung sections. The architecture of engorged cervical lymph nodes in *Ncr1^gfp/gfp^* mice was assessed following hematoxylin-eosin staining of fixed sections.

Assessment of lung macrophage content and interleukin-23 (IL-23) production was performed by immunofluorescent staining of fixed lung sections. Sections of formalin-fixed, paraffin-embedded mouse lung tissue (5 μm) were cut onto glass slides, deparaffinized in xylene, and rehydrated in graded ethanol changes (100, 95, 80, and 70%). Antigen retrieval was performed by incubating sections in citrate buffer containing 10 mM sodium citrate and 0.05% Tween-20 (pH 6.0) at 95°C in a microwave for 10 min. Slides were then cooled to room temperature, nonspecific protein binding was blocked by incubating with 1% BSA in PBS for 30 min, and excess autofluorescence was blocked by incubating with 0.1% Sudan Black B (Sigma Cat. No. 199664) in 70% ethanol for 20 min. Slides were then incubated with primary antibodies (rat anti-mouse F4/80, BioLegend Cat. No. 123101, 1:400 and rabbit anti-mouse IL-23 Thermo Fisher Cat. No. PA5-20239, 1:500) overnight at 4°C. Staining was revealed using species-specific fluorescently conjugated secondary antibodies (anti-rabbit AF647 Cat. No. A31573 and anti-rat AF594 Cat. No. A21209; Invitrogen). Nuclei were detected by staining with DAPI (BioLegend Cat. No. 422801). Images were acquired using a Zeiss Axio-M1 epifluorescence or Leica confocal microscope. Nucleated F4/80^+^ and IL-23^+^ cells were manually quantified using the Cell Counter tool in ImageJ (National Institutes of Health). Cell numbers were quantified from a total of 5 high-power fields per mouse.

### Quantitative PCR

Snap-frozen lung tissue was homogenized in TRIzol. Purified RNA was loaded on a purification column and treated with DNase (both Qiagen) to remove residual genomic DNA. Quantitative PCR was conducted using the primers detailed in Table 1.

**Table 1.** Quantitative PCR primer sequences

Gene	Forward Primer	Reverse Primer
*VEGFa*	CAGGCTGCTGTAACGATGAA	CTATGTGCTGGCTTTGGTGA
*VEGFc*	GGGAAGAAGTTCCACCATCA	ATGTGGCCTTTTCCAATACG
*Angiopoietin-1*	GGGGGAGGTTGGACAGTAA	CATCAGCTCAATCCTCAGC
*Angiopoietin-2*	CTGTGCGGAAATCTTCAAGTC	TGCCATCTTCTCGGTGTT
*Il6*	GATGGATGCTACCAAACTGGAT	CCAGGTAGCTATGGTACTCCAGA
*Il17a*	CAGGGAGAGCTTCATCTGTGT	GCTGAGCTTTGAGGGATGAT
*Ifng*	ATCTGGAGGAACTGGCAAAA	TTCAAGACTTCAAAGAGTCTGAGG
*Il21*	GACATTCATCATTGACCTCGTG	TCACAGGAAGGGCATTTAGC
*Il22*	TGACGACCAGAACATCCAGA	AATCGCCTTGATCTCTCCAC
*Il23a p19*	TCCCTACTAGGACTCAGCCAAC	AGAACTCAGGCTGGGCATC
*Rpl32*	AAGCGAAACTGGCGGAAAC	TAACCGATGTTGGGCATCAG

VEGF, vascular endothelial growth factor.

### Western Blot Analysis

Protein was isolated from homogenized lung tissue using a lysis buffer consisting of 50 mM Tris·HCl (pH 7.4–7.6), 150 mM NaCl, 1% Igepal CA-630, 10 mM NaF, 0.5% sodium deoxycholate, 2 mM sodium orthovanadate (all Sigma), and a protease inhibitor cocktail (Roche). Lysed protein was quantified using the Lowry method (Bio-Rad), run on SDS-PAGE, and transferred to Hybond PVDF membrane (GE Healthcare). Immunoblotting was performed using primary antibodies to mouse angiopoietin-2 (clone: F-18, Santa Cruz, Dallas, TX) and α-tubulin (clone: DM1A, Sigma-Aldrich, St. Louis, MO), horseradish peroxidase-conjugated anti-rabbit or anti-mouse secondary antibodies (both Dako), and visualized using ECL Plus chemiluminescent reagent (GE Healthcare). The specificity of the angiopoietin-2 antibody was validated previously by Abbott and Buckalew (1).

### Statistical Analysis

All data were collated in GraphPad Prism 6, which was also used for statistical analysis. All data are presented as means ± SE. Assessment of significance between two groups was performed by unpaired, two-tailed Student’s *t*-test. For multiple comparisons, significance was assessed by 1- or 2-way ANOVA, followed by the appropriate post hoc test, as indicated in the figure legends. A *P* value < 0.05 was considered significant.

## RESULTS

### 

#### NK cell-deficient Nfil3^−/−^ mice develop PH with age.

**Fig. 1.**
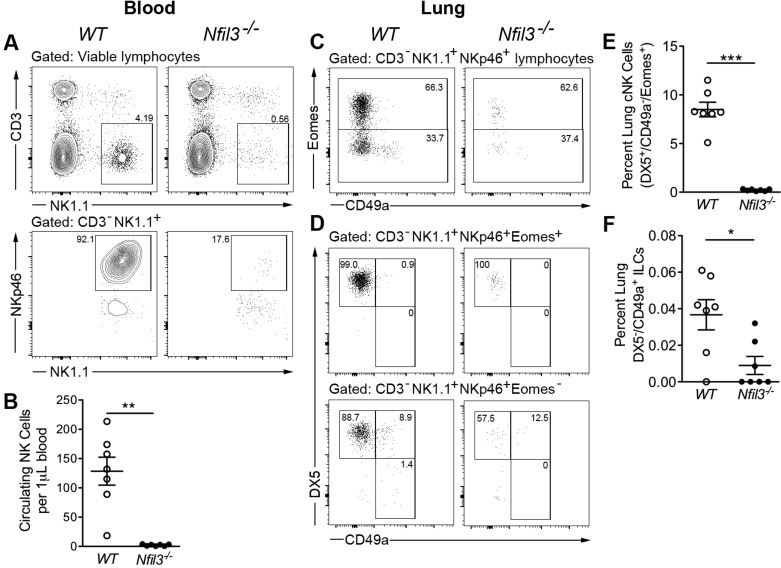
*Nfil3^−/−^* mice are grossly deficient in natural killer (NK) and ILC1 cells. *A*: representative flow cytometry plots showing the gating of CD3^−^NK1.1^+^NKp46^+^ NK cells within the circulation of 8–10-wk-old wild-type (WT) and *Nfil3^−/−^* mice. *B*: absolute quantification of circulating NK1.1^+^NKp46^+^ NK cells (*n* = 7). *C* and *D*: representative flow cytometry plots showing the gating of NK and ILC1 populations within the lungs of WT and *Nfil3^−/−^* mice. *E*: quantification of lung conventional NK (cNK) cells. *F*: quantification of lung CD49a^+^DX5^−^ ILC1 cells (*n* = 7). Statistical significance was determined using the Mann-Whitney test. *P < 0.05, **P < 0.01, and ***P < 0.001. Means ± SE. ILC, innate lymphoid cell.

Cytometric analysis of peripheral blood from *Nfil3^−/−^* mice and WT littermate controls confirmed the severe attenuation of absolute circulating NK cell numbers in mice lacking the NFIL3 transcription factor (Fig. 1, *A* and *B*). This reduction was also reflected in the lungs of *Nfil3^−/−^* mice, whereas DX5^+^/CD49a^−^/Eomes^+^ NK cells, which are equivalent to conventional circulating NK cells, were significantly reduced relative to WT littermates (Fig. 1, *C*–*E*). In addition to this NK cell deficiency, the loss of NFIL3 has also been shown to impact ILC populations (18, 20, 21), including DX5^−^/CD49a^+^/Eomes^−^ ILC1 cells, which represented only a small proportion of total NK1.1^+^/NKp46^+^ cells in the lung (Fig. 1, *C*, *D*, and *F*). In contrast to previous reports identifying NFIL3-independent DX5^−^/CD49a^+^ trNK cells or ILC1s in the liver and uterus (7, 43), this population was markedly depleted in the lungs of *Nfil3^−/−^* mice.

**Fig. 2.**
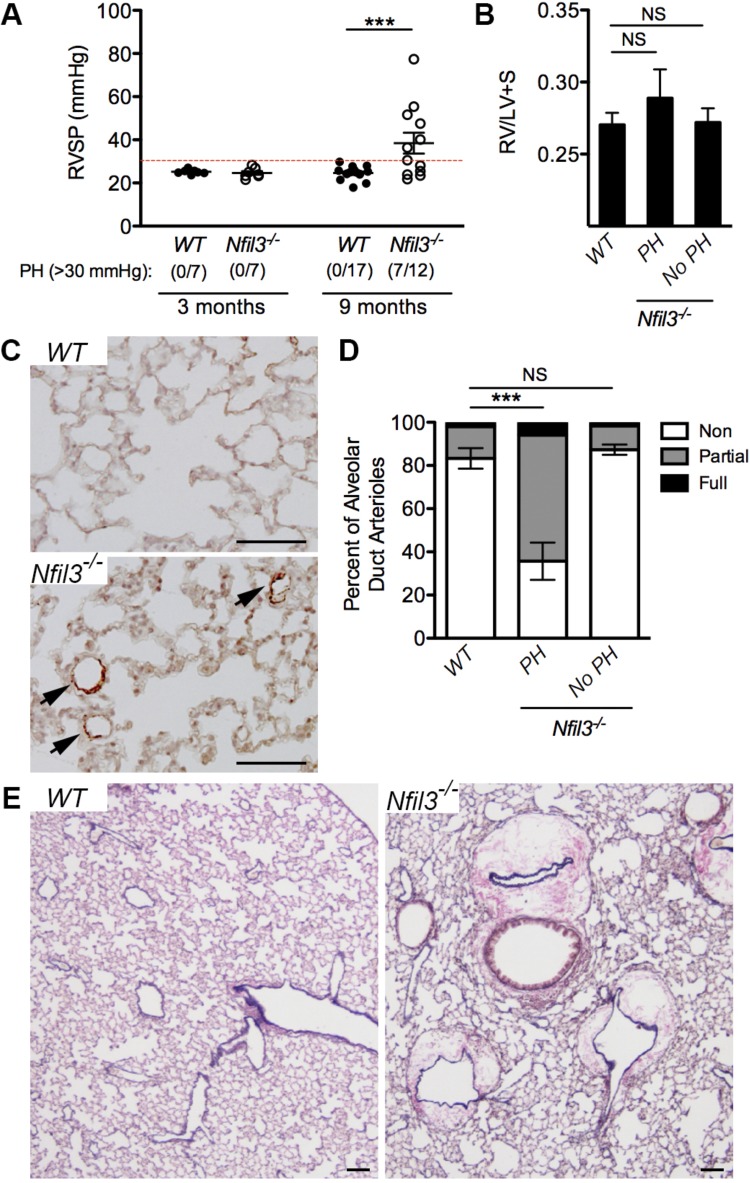
A subset of *Nfil3^−/−^* mice develop pulmonary hypertension with age. *A*: assessment of right ventricular systolic pressure (RVSP) in naive *Nfil3^−/−^* mice and wild-type (WT) littermate controls at 3 mo (*n* = 7 per group) and 9 mo (*n* = 17 for WT, *n* = 12 for *Nfil3^−/−^*). The onset of pulmonary hypertension (PH) was defined as an RVSP >30 mmHg. Statistical significance was determined using Fisher’s exact test. *B*: right ventricular hypertrophy (Fulton index, RV/LV + S = ratio of RV weight over LV and septal weight) in 9-mo-old *Nfil3^−/−^* mice with and without PH and WT littermate controls (1-way ANOVA, Dunnett’s posttest). *C*: immunohistochemical assessment of alveolar duct arteriole muscularization in the mice described in *B*, as determined by staining lung sections for smooth muscle α-actin. Arrows indicate muscularized alveolar duct arterioles. *D*: quantitative assessment of pulmonary arterial muscularization in the mice described in *B*, divided into WT controls (*n* = 13), *Nfil3^−/−^* with spontaneous PH (*n* = 6), and *Nfil3^−/−^* without spontaneous PH (*n* = 5). Displayed as non-, partially, and fully muscularized arterioles as a percentage of total alveolar duct arterioles (1-way ANOVA, Dunnett’s posttest for nonmuscularized vessels vs. WT controls). *E*: elastic van Gieson staining of fixed lung sections from 9-mo-old *Nfil3^−/−^* mice and WT littermate controls showing substantial cuffing around the pulmonary arteries and veins of aged *Nfil3^−/−^* mice. Scale bars = 100 μm. ***P < 0.001. Means ± SE. LV, left ventricle; NS, not significant.

Assessment of cardiopulmonary function by closed-chest conductance catheterization of the right ventricle revealed no elevation of RVSP in 3-mo-old *Nfil3^−/−^* mice when compared with WT littermate controls (Fig. 2*A*). However, by 9 mo of age, a significant proportion of *Nfil3^−/−^* mice developed a spontaneous form of PH, as exemplified by an RVSP greater than 30 mmHg (Fig. 2*A*). Similar elevations in RVSP were not observed in any of the aged WT littermate controls. Although PH in aged *Nfil3^−/−^* mice was not accompanied by significant RV hypertrophy, as measured by a ratio of RV versus LV and septal weight (Fig. 2*B*), elevated RVSP in these animals was associated with a significant muscularization of pulmonary alveolar duct arterioles (Fig. 2, *C* and *D*). This muscularization of the pulmonary arterioles was not observed in either WT littermate controls or the subset of *Nfil3^−/−^* mice that failed to develop PH with age (RVSP <30 mmHg). A secondary examination of lung structure in the aged *Nfil3^−/−^* mice by elastic van Gieson staining also revealed substantial cuffing around the pulmonary arteries and veins, suggesting perivascular edema and reduced endothelial barrier function in these animals (Fig. 2*E*).

#### Spontaneous PH in Nfil3^−/−^ mice is not secondary to left heart failure.

**Fig. 3.**
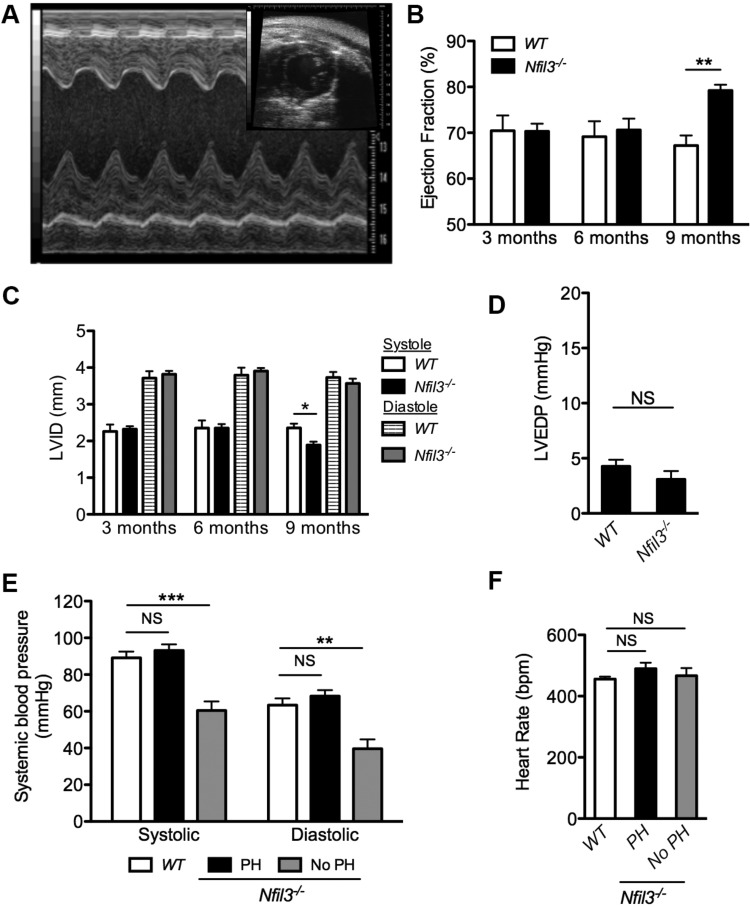
Spontaneous pulmonary hypertension (PH) in *Nfil3^−/−^* mice is not secondary to left heart failure. Representative transthoracic echocardiography images (*A*) showing a short axis view of the left ventricle (*inset*) and the corresponding M-mode sample. Longitudinal assessment of ejection fraction (*B*) and left ventricular internal dimension (LVID) (*C*) at systole and diastole in naive *Nfil3^−/−^* mice and wild-type (WT) littermate controls at 3, 6, and 9 mo of age (*n* = 8, significance assessed by 2-way ANOVA). Quantification of left ventricular end-diastolic pressure (LVEDP) (*D*) in aged WT controls (*n* = 12) and *Nfil3^−/−^* mice (*n* = 8) by closed-chest conductance catheterization. Systemic blood pressure measurements (*E*) were collected from the aortic arch of aged WT littermate controls (*n* = 14) and *Nfil3^−/−^* mice, which were divided based on the presence (*n* = 6) or absence (*n* = 4) of PH, as defined by a right ventricular systolic pressure >30 mmHg (1-way ANOVA, Dunnett’s posttest vs. WT controls). Anaesthetized heart rates (*F*) corresponding to the measurement of systemic blood pressure in aged *Nfil3^−/−^* mice, as described in *E* (1-way ANOVA, Dunnett’s posttest vs. WT controls). **P < 0.01 and ***P < 0.001. Means ± SE. NS, not significant.

Since PH can manifest as a secondary consequence of left-sided heart failure, a full assessment of LV function was also performed on the aged *Nfil3^−/−^* and WT mice that underwent RV assessment. Longitudinal transthoracic echocardiography identified no differences in LV EF in *Nfil3^−/−^* mice at 3 and 6 mo of age when compared with WT controls (Fig. 3, *A* and *B*), which agrees with the absence of a disease phenotype in younger animals. However, by 9 mo, the development of PH in aged *Nfil3^−/−^* mice resulted in a hyperdynamic left ventricle, as demonstrated by an increase in LV EF. An assessment of LV internal dimension at systole (LVIDs) and diastole (LVIDd) revealed that this elevated EF was due to increased contraction at systole and was not the result of changes in the diastolic diameter of the left ventricle (Fig. 3*C*).

 Catheterization of the left heart revealed no difference in LVEDP between aged *Nfil3^−/−^* mice and WT littermate controls (Fig. 3*D*), further confirming that PH in these animals was not secondary to LV failure. Aged *Nfil3^−/−^* mice with PH (RVSP >30 mmHg) also exhibited normal systemic blood pressure when compared with WT controls (Fig. 3*E*). However, an assessment of systemic blood pressure in the subset of *Nfil3^−/−^* mice that did not develop elevated RVSP with age identified a significant reduction of both systolic and diastolic blood pressure in these animals. An examination of anaesthetized heart rates did not identify a significant difference in mean heart rate between groups, indicating that this reduction was not the product of differential sedation (Fig. 3*F*). Instead, these results suggest that vascular dysfunction in the subset of aged *Nfil3^−/−^* mice with seemingly normal RVSP extends to the systemic circulation, causing systemic hypotension in these animals.

#### Loss of the NK-activating receptor NKp46 causes pulmonary and systemic vascular dysfunction.

Although results from the aged *Nfil3^−/−^* mice provide compelling evidence to support a role for NK cells in the regulation of both pulmonary and systemic arterial pressure, loss of the NFIL3 transcription factor can impact a variety of immune populations, including ILCs (17) and dendritic cells (22), as well as nonimmune processes, such as circadian clock and insulin processing (30, 47). To address this limitation and pinpoint the precise role of NK cells in vascular homeostasis, pulmonary and systemic vascular function were also assessed in *Ncr1-Gfp* mice as an independent genetic model of NK cell dysfunction.

**Fig. 4.**
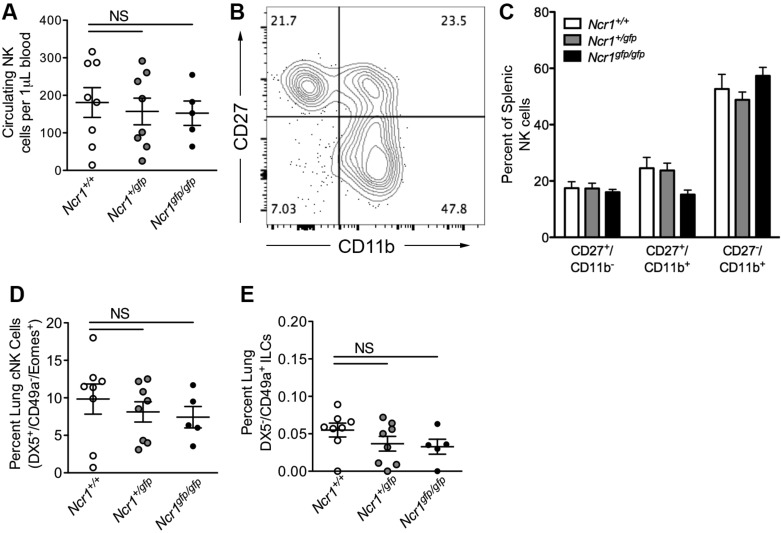
Assessment of natural killer (NK) and innate lymphoid cell (ILC) subsets in the blood and lungs of *Ncr1-Gfp* mice. *A*: absolute quantification of circulating CD3^−^NK1.1^+^ NK cells in *Ncr1^+/+^*, *Ncr1^+/gfp^*, and *Ncr1^gfp/gfp^* mice (*n* = 5–8). *B*: representative flow cytometry plot showing the stages of splenic NK cell maturity, as determined by surface expression of CD27 and CD11b. *C*: quantification of NK cell maturation in the spleens of *Ncr1^+/+^*, *Ncr1^+/gfp^*, and *Ncr1^gfp/gfp^* mice (*n* = 3–7). *D*: quantification of lung conventional NK cells. *E*: quantification of lung CD49a^+^DX5^−^ ILC1 cells (*n* = 5–8). All analyses consisted of one-way ANOVA, Dunnett’s posttest vs. *Ncr1^+/+^* controls. Means ± SE. NS, not significant.

Mice lacking one (*Ncr1^+/gfp^*) or two (*Ncr1^gfp/gfp^*) copies of *Ncr1* exhibited a normal cohort of peripheral NK cells, as measured by total circulating NK cell number (Fig. 4*A*), and unimpaired NK cell maturation, as quantified by the progression of immature (CD11b^−^CD27^+^) NK cells to intermediate (CD11b^+^CD27^+^) and mature (CD11b^+^CD27^−^) phenotypes (11) (Fig. 4, *B* and *C*). A further examination of NKp46-expressing pulmonary lymphocyte populations demonstrated equivalent levels of conventional, DX5^+^/CD49a^−^/EOMES^+^ NK cells (Fig. 4*D*), and DX5^−^/CD49a^+^/EOMES^−^ ILC1 cells (Fig. 4*E*) in the lungs of *Ncr1^+/gfp^*, *Ncr1^gfp/gfp^*, and WT littermates, indicating that the loss of NKp46 on these populations does not result in gross differences in cell number.

**Fig. 5.**
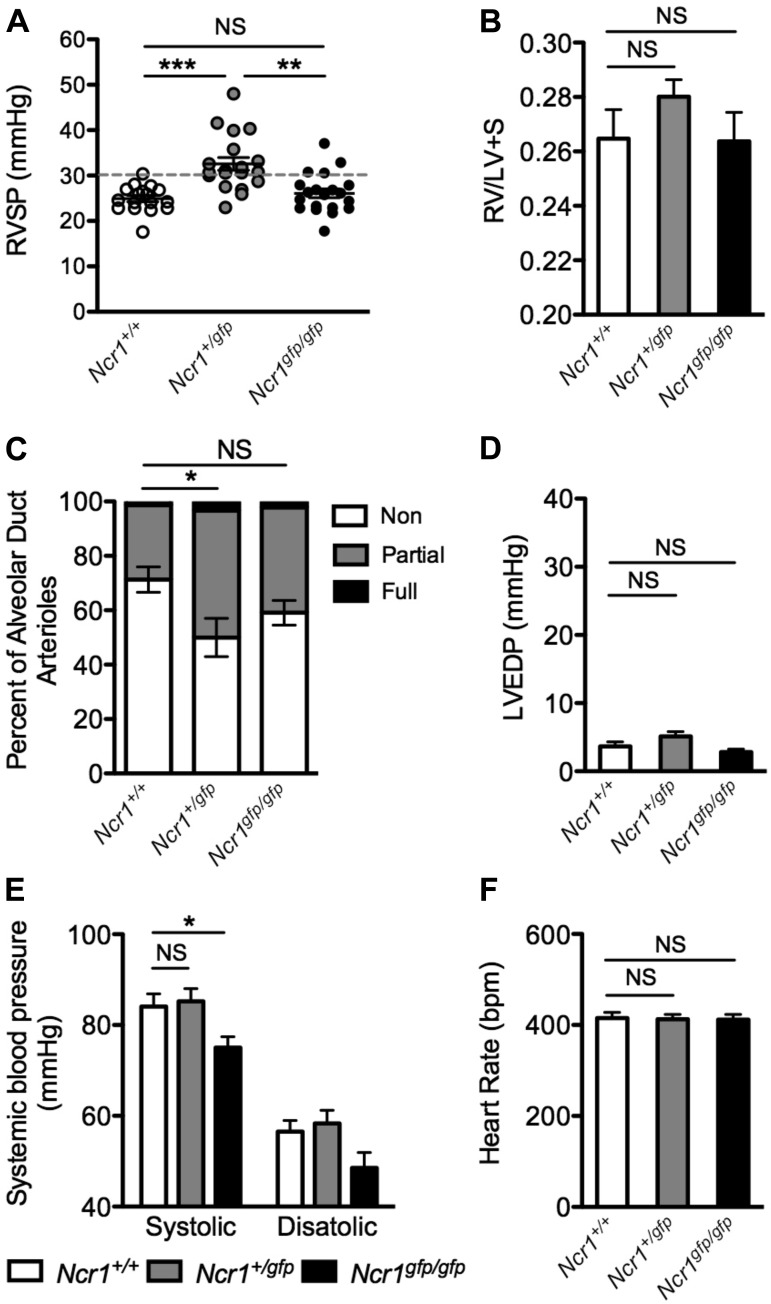
Pulmonary and systemic vascular dysfunction in *Ncr1-Gfp* mice. *A*: assessment of right ventricular systolic pressure (RVSP) in naïve and wild-type (WT) controls (*Ncr1^+/+^*), *Ncr1^+/gfp^* mice, and *Ncr1^gfp/gfp^* mice at 4 mo of age (*n* = 17–20; 1-way ANOVA, Tukey’s posttest vs. *Ncr1^+/+^* controls). *B*: quantification of right ventricular hypertrophy in the mice specified in *A* (*n* = 20–22). *C*: quantitative assessment of pulmonary arterial muscularization in the mice described in *A*, displayed as non-, partially, and fully muscularized arterioles as a percentage of total alveolar duct arterioles (*n* = 6–7; 1-way ANOVA, Dunnett’s posttest for nonmuscularized vessels vs. WT controls). *D*: quantification of left ventricular end-diastolic pressure (LVEDP) in WT, *Ncr1^+/gfp^*, and *Ncr1^gfp/gfp^* mice (*n* = 12–13; 1-way ANOVA, Dunnett’s posttest vs. WT controls). *E*: systemic blood pressure measurements from the aortic arch of the mice specified in *A* (*n* = 12–13; 1-way ANOVA, Dunnett’s posttest vs. WT controls). *F*: corresponding anaesthetized heart rates from the aortic arch of the mice specified in *A* (*n* = 12–13; 1-way ANOVA, Dunnett’s posttest vs. WT controls). *P < 0.05, **P < 0.01, and ***P < 0.001. Means ± SE. NS, not significant.

An assessment of cardiopulmonary function revealed a spontaneous elevation of RVSP in heterozygous null *Ncr1^+/gfp^* mice by 4 mo of age (Fig. 5*A*), several months before the spontaneous onset of PH in the *Nfil3^−/−^* strain. As with spontaneous disease in the *Nfil3^−/−^* mice, RV hypertrophy in *Ncr1^+/gfp^* mice was not significant in comparison with WT controls (Fig. 5*B*). However, PH in these animals was accompanied by significant muscularization of alveolar duct arterioles (Fig. 5*C*). Examination of left heart function in *Ncr1^+/gfp^* mice demonstrated that PH in these animals was not associated with elevated LVEDP (Fig. 5*D*) or changes in systemic blood pressure when compared with WT littermates (Fig. 5, *E* and *F*).

Interestingly, spontaneous PH was not observed in *Ncr1^gfp/gfp^* mice, which completely lack the gene encoding NKp46. *Ncr1^gfp/gfp^* animals did not develop significantly elevated RVSP (Fig. 5*A*), RV hypertrophy (Fig. 5*B*), or muscularization of their pulmonary arterioles (Fig. 5*C*). Instead, *Ncr1^gfp/gfp^* mice exhibited a significant reduction in systemic blood pressure (Fig. 5*E*), mirroring the phenotype observed in the subset of aged *Nfil3^−/−^* mice that failed to develop PH. As with the *Nfil3^−/−^* mice, heart rates were similar between *Ncr1^+/+^*, *Ncr1^+/gfp^*, and *Ncr1^gfp/gfp^* littermates, indicating that systemic hypotension in *Ncr1^gfp/gfp^* mice was not a consequence of differential sedation (Fig. 5*F*).

#### Enhanced IL-23 production in the lungs of Ncr1-Gfp and aged Nfil3^−/−^ mice.

**Fig. 6.**
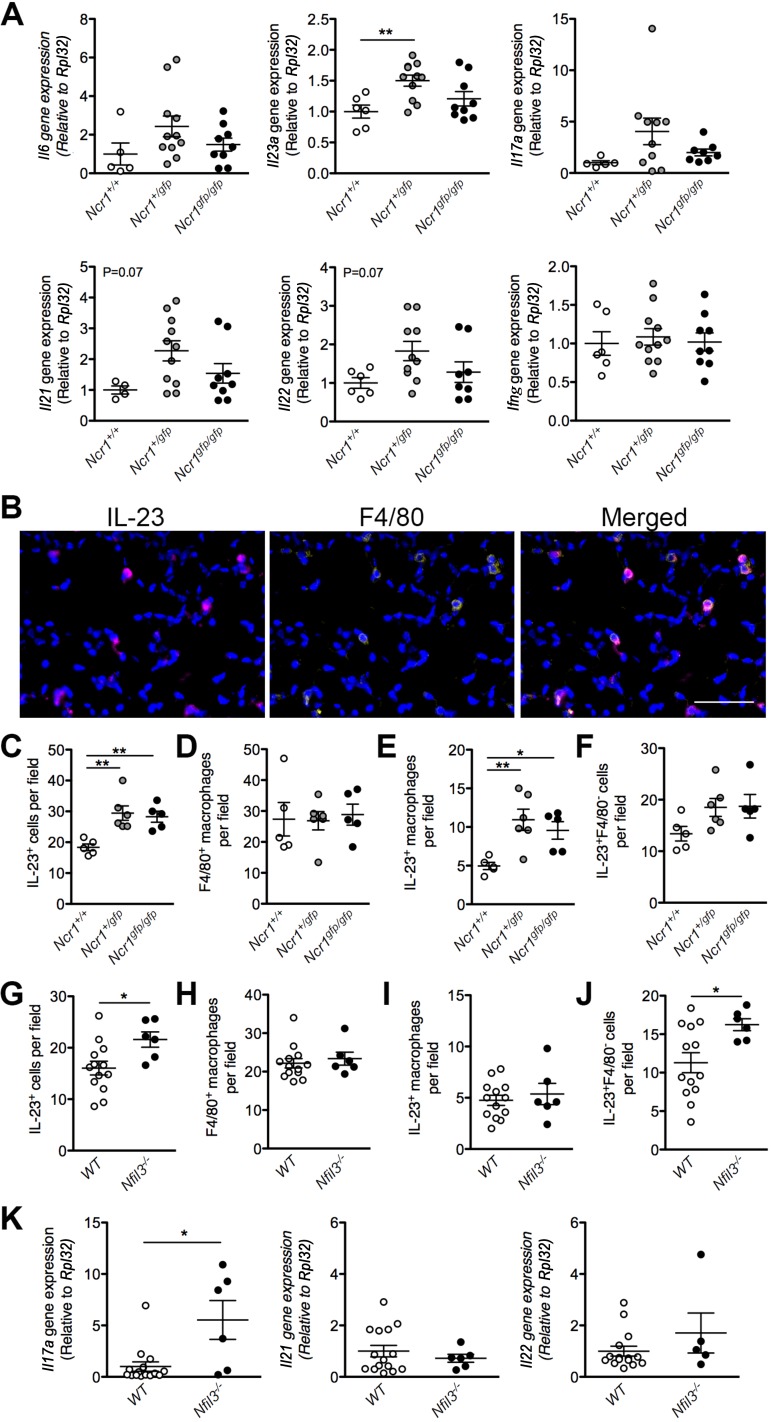
Increased IL-23 production and downstream cytokine expression in the *Ncr1-Gfp* and *Nfil3^−/−^* mouse models of natural killer cell impairment and deficiency. Assessment of inflammatory gene expression in the lungs of *Ncr1^+/+^*, *Ncr1^+/gfp^*, and *Ncr1^gfp/gfp^* mice (*A*), normalized to the *Rpl32* reference gene (*n* = 4–11; 1-way ANOVA, Dunnett’s posttest vs. *Ncr1^+/+^* controls). Representative immunofluorescent staining (*B*) of mouse lung tissue for IL-23 (magenta), macrophages (F4/80, yellow), and nuclei (DAPI, blue). Scale bar = 50 μm. Quantification of IL-23^+^ cells (*C* and *G*), F4/80^+^ macrophages (*D* and *H*), IL-23^+^ macrophages (*E* and *I*), and IL-23^+^F4/80^−^ cells (*F* and *J*) in the lungs of both *Ncr1-Gfp* mice (*C*–*F*) (*n* = 5–6; 1-way ANOVA, Dunnett’s posttest vs. *Ncr1^+/+^* controls) and *Nfil3^−/−^* mice (*G*–*J*) with pulmonary hypertension or WT littermate controls (*n* = 6–13; Student’s *t*-test). Expression of Th17-associated cytokines (*K*) in the lungs of *Nfil3^−/−^* mice with pulmonary hypertension and WT littermate controls (*n* = 5–13; Student’s *t*-test). *P < 0.05 and **P < 0.01. Means ± SE.

In an attempt to provide some insight into the mechanisms underlying the development of PAH in our models of NK cell impairment, the lungs of *Ncr1^+/+^*, *Ncr1^+/gfp^*, and *Ncr1^gfp/gfp^* mice were screened for inflammatory gene expression (Fig. 6*A*). This screen, which focused primarily on cytokines involved in Th17-type responses, identified increased expression of the gene encoding IL-23 in the lungs of *Ncr1^+/gfp^* mice relative to WT controls. In addition to this increase, our data also suggest an elevation of other cytokines of this pathway in association with the development of disease, particularly IL-17A, IL-21, and IL-22. However, changes in these genes did not reach significance. No differences were observed in IFNγ, which has previously been linked to the NK-mediated development of systemic hypertension (23).

An immunofluorescent analysis of lung tissues confirmed a significant increase in IL-23^+^ cells in the lungs of both *Ncr1^+/gfp^* and *Ncr1^gfp/gfp^* mice relative to controls (Fig. 6*C*). Costaining with the macrophage marker F4/80 identified macrophages as the primary source of increased IL-23 in these animals (Fig. 6, *D* and *E*). Although no differences were observed in total lung macrophage counts between mice of each genotype (Fig. 6*D*), the number of IL-23^+^ macrophages was more than doubled in the *Ncr1^+/gfp^* mice when compared with WT controls (Fig. 6*E*). An increase in IL-23^+^ macrophages was also observed in *Ncr1^gfp/gfp^* mice. However, in line with our hemodynamic and gene expression data, this increase was more modest than what was observed in the heterozygous animals. The number of F4/80 negative cells expressing IL-23 also appeared to be increased in both *Ncr1^+/gfp^* and *Ncr1^gfp/gfp^* mice (Fig. 6*F*). However, differences in this cellular subset, which likely include other myeloid cell types, such as dendritic cells, did not reach statistical significance.

Importantly, a similar increase in IL-23^+^ cells was also identified in the lungs of *Nfil3^−/−^* mice that developed PH with age (Fig. 6*G*), suggesting the possibility of a common inflammatory mechanism of disease in both mouse models of NK cell insufficiency. However, in the *Nfil3^−/−^* animals, this increase was not associated with elevated IL-23^+^ macrophages but was instead attributable to changes in the IL-23^+^F4/80^−^ cellular subset (Fig. 6, *H*–*J*). Assessment of downstream inflammatory gene expression in these mice identified a significant increase in the gene encoding IL-17A but not IL-21 or IL-22 (Fig. 6*K*), providing a link between PH and Th17-associated inflammation in this model.

#### Altered lymphatic phenotype in Ncr1-Gfp mice.

**Fig. 7.**
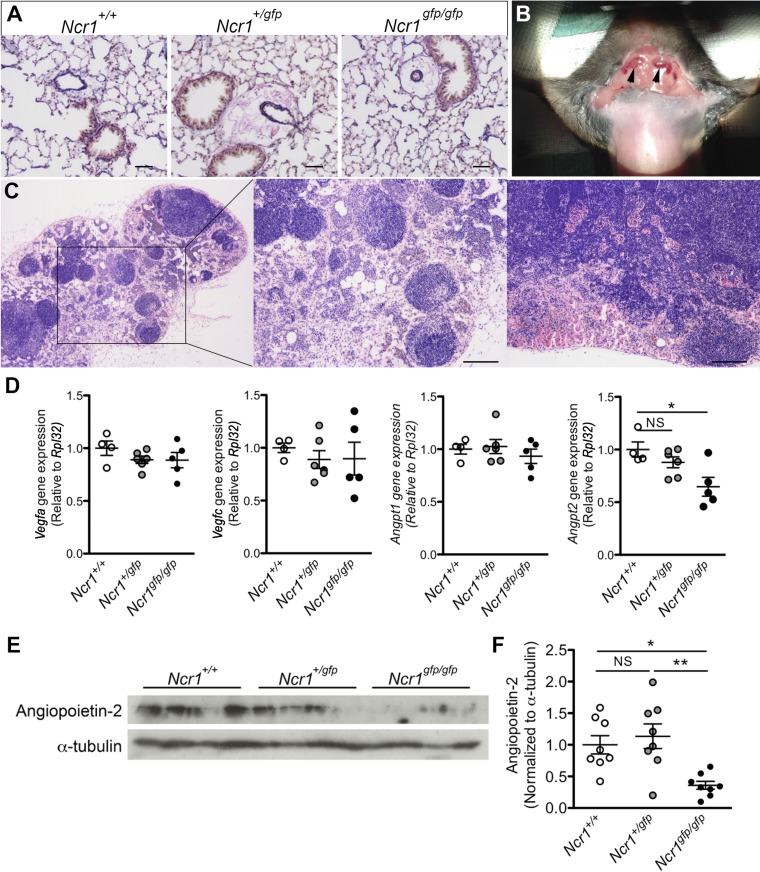
Altered lymphatic function and angiogenic gene expression in *Ncr1-Gfp* mice. *A*: representative images of elastic van Gieson-stained fixed-lung sections from *Ncr1^+/+^*, *Ncr1^+/gfp^*, and *Ncr1^gfp/gfp^* mice showing perivascular edema around the pulmonary vasculature of both *Ncr1^+/gfp^* and *Ncr1^gfp/gfp^* mice. Scale bars = 100 μm. *B*: image showing enlarged cervical lymph nodes (arrows) in an *Ncr1^gfp/gfp^* mouse. *C*: representative hematoxylin-eosin staining of enlarged lymph nodes from *Ncr1^gfp/gfp^* mice. Scale bars = 200 μm. *D*: assessment of angiogenic gene expression the lungs of *Ncr1^+/+^*, *Ncr1^+/gfp^*, and *Ncr1^gfp/gfp^* mice, normalized to the *Rpl32* reference gene (*n* = 4–6). *E*: representative immunoblot of angiopoietin-2 protein in the lungs of the mice detailed in *A* (*n* = 8). *F*: quantification of angiopoietin-2 protein in the lungs of the mice detailed in *A* (*n* = 8). *P < 0.05 and **P < 0.01, as determined by a 1-way ANOVA with a Tukey’s posttest. Means ± SE.

Our analysis of inflammatory gene expression does not explain the absence of PH in *Ncr1^gfp/gfp^* mice or the observation of low systemic blood pressure in these animals. Although no significant pulmonary arteriolar muscularization was observed in *Ncr1^gfp/gfp^* mice, substantial vascular cuffing was observed in the lungs of both *Ncr1^+/gfp^* and *Ncr1^gfp/gfp^* animals, which suggests the presence of perivascular edema in the lungs of animals of both genotypes (Fig. 7*A*). Under macroscopic examination, a subset of mice bearing the *Ncr1^gfp/gfp^* genotype was also found to exhibit markedly enlarged superficial cervical lymph nodes (Fig. 7*B*). Assessment of enlarged nodes revealed substantial lymphangiogenesis and RBC infiltration (Fig. 7*C*), further supporting a phenotype of impaired endothelial barrier integrity in the systemic circulation of these animals.

In addition to the inflammatory genes detailed above, lung tissues from *Ncr1-Gfp* mice and WT littermates were examined for the expression of vascular endothelial growth factor (VEGF)a, VEGFc, and the angiopoietins, which are known to influence angiogenesis, vascular integrity, and lymphatic endothelial function and have been implicated in NK-mediated uterine vascular remodeling during pregnancy (3, 19, 26, 29). This screen identified reduced angiopoietin-2 (*Angpt2*) gene expression accompanying the complete loss of *Ncr1* (Fig. 7*D*). The reduction was confirmed by immunoblotting, which identified significantly reduced angiopoietin-2 protein exclusively in the lungs of *Ncr1^gfp/gfp^* animals (Fig. 7, *E* and *F*). A subsequent examination of gene expression in isolated NK cells revealed that these cells do not express angiopoietin-2, independent of *Ncr1* genotype (data not shown), indicating that the loss of angiopoietin-2 in the lungs is likely due to reduced expression by another cell type, such as the vascular endothelium (46). Together, these findings provide mechanistic insight that links NK cell impairment to a signaling pathway that is known to regulate both vascular and lymphatic structure and integrity.

## DISCUSSION

We report the spontaneous development of PH in two genetic models of NK cell insufficiency, NK cell-deficient *Nfil3^−/−^* mice and *Ncr1^+/gfp^* mice, which are heterozygous for the NK activating receptor NKp46. In both models, PH was observed in the absence of elevated LVEDP, indicating that the increases in RV pressures were not secondary to venous congestion resulting from left heart failure. In contrast, our assessment of systemic blood pressure identified abnormalities exclusively in mice with seemingly normal pulmonary vascular pressures, including the subset of aged *Nfil3^−/−^* mice that failed to develop elevated RVSP with age and *Ncr1^gfp/gfp^* mice, which completely lack NKp46. Both of these groups exhibited a marked reduction in systemic blood pressure, suggesting a model whereby moderate NK cell dysfunction can result in PH, whereas severe NK cell impairment causes vascular dysfunction that extends to the systemic circulation, resulting in reduced systemic blood pressure and preventing the manifestation of elevated pressures in the pulmonary circulation.

Importantly, our identification of similar phenotypes in two independent genetic models of NK cell impairment serves to address the limitations associated with the broad activity of the NFIL3 transcription factor in a range of immune and nonimmune processes (17, 22, 30, 47). Unlike NFIL3, the loss of NKp46 in *Ncr1-Gfp* mice is more strictly limited to the narrow range of cell types expressing this receptor, including NK cells, ILC1 cells, and NKp46^+^ ILC3 cells, which are not typically present in the lungs of naïve mice and are instead more prominent in other tissues, including the gut and uterus (12, 17). Our report of spontaneous PH in *Ncr1^+/gfp^* mice, as well as systemic hypotension in the more severely impaired *Ncr1^gfp/gfp^* animals, strongly supports the hypothesis that the vascular defects in the NK-deficient mouse models examined here are the direct result of the impact of these genetic manipulations on NK or ILC1 populations. Although our use of two genetic models adds to the rigor of our findings, these models did not include the depletion of NK cells in adult mice. As such, we cannot rule out the potential impact of a developmental defect on pulmonary or systemic vascular function. Future studies examining the conditional ablation and adoptive transfer of NK cells will help to confirm the importance of these cells in disease.

Our work builds on previous reports highlighting a role for NK cells in the regulation of vascular structure and integrity. Studies examining uterine vascular remodeling in *Nfil3^−/−^* mice during pregnancy identified the persistent muscularization of uterine arterial walls in pregnant *Nfil3^−/−^* mice when compared with WT littermates, resulting in the reduced fetal weight of offspring from litters of equivalent size (7). While these findings support the now broadly appreciated role for decidual NK cells in vascular remodeling during pregnancy, to date, few studies have addressed the capacity of NK cells to influence vascular beds outside the uterus (23, 49). The current work complements a previous report demonstrating a critical role for NK cells in the prevention of pulmonary vascular hyperpermeability following acute myocardial infarction (49). This report is particularly relevant to our study, as the lung perivascular edema observed in both *Ncr1-Gfp* and aged *Nfil3^−/−^* mice indicates that disease phenotypes in these animals could be the result of impaired endothelial barrier function. Loss of endothelial barrier integrity is a prominent feature of PAH, both in humans and animal models of disease (27, 37, 50). Importantly, an examination of hyperpermeability in the hypoxia-SU5416 rat model of PAH localized impaired endothelial barrier function to perivascular fluid cuffs around extra alveolar vessels and not the alveolar capillaries (50), a pattern that is similar to what we report in the *Nfil3^−/−^* and *Ncr1-Gfp* mice. The observation of engorged cervical lymph nodes in *Ncr1^gfp/gfp^* mice provides further evidence supporting vascular hyperpermeability in these animals, highlighting the extension of vascular dysfunction to the systemic circulation in the context of more severe NK cell impairment.

Our identification of increased IL-23 production in the lungs of our pulmonary hypertensive mouse models provides novel mechanistic insight into the processes by which NK cell deficiency might be inducing changes in the pulmonary circulation. To our knowledge, our findings are the first time that IL-23 has been linked to the development of PAH. These findings also align with recent reports identifying the importance of the IL-6/Th17 axis to the development of PH in mice exposed to chronic hypoxia (20). IL-23 is known to synergize with IL-6 and TGFβ to drive naïve T cells toward a Th17 phenotype and the production of cytokines, including IL-17A/F, IL21, and IL-22 (51). Although IL-17-, IL-21-, and IL-22-mediated inflammation is typically associated with the actions of these Th17 cells, innate lymphocytes, including NK cells, γδT cells, and ILC subsets, have also been shown to act as a major source of these cytokines in mice and humans (9, 34, 45). Future studies will focus on confirming a role for IL-23 in the pathogenesis of disease, as well as examining the potential contribution of IL-17, IL-21, and IL-22 to the development of PAH in the context of NK cell deficiency. This work will also examine the mechanisms by which NK cell loss or impairment might lead to the enhanced production of IL-23 by pulmonary macrophages and other myeloid cell types. Although previous studies demonstrated an ability of NK cells to “edit” mature dendritic cell populations (13), there is currently little information on how NK-macrophage cross talk might influence macrophage phenotype and cytokine production in the lung.

The mechanisms by which severe NK cell deficiency or impairment lead to reduced systemic blood pressure remain uncertain. This finding calls to mind Castleman’s disease, which is defined by lymph node hyperplasia and has been reported in association with both PAH (8, 28) and systemic hypotension (2, 25). However, whereas Castleman’s is often characterized by elevated IL-6 production, our studies found no such elevation in the *Ncr1^gfp/gfp^* group that exhibited enlarged lymph nodes and low systemic blood pressure. These mice also did not exhibit excessive macrophage accumulation in the lung or inflammatory infiltrates in other tissues that could explain a drop in systemic blood pressure, only a change in the propensity of lung macrophages to produce IL-23.

In contrast, the identification of reduced angiopoietin-2 gene and protein expression in the lungs of *Ncr1^gfp/gfp^* mice provides a potentially interesting mechanistic link between NK cell impairment and a signaling pathway that is known to regulate vascular structure, integrity, and tone. Circulating angiopoietin-2 levels are elevated in individuals with systemic hypertension (10) and angiopoietin-1/angiopoietin-2 ratios are reduced in pregnant women who develop preeclampsia (4). Angiopoietin-2 is also linked to the regulation of vascular integrity and lymphatic development. Transgenic mice overexpressing angiopoietin-2 demonstrate attenuated LPS-induced pulmonary vascular leak (40). Moreover, angiopoietin-2 knockout mice exhibit altered lymphatic development, which could help to explain our observations in the *Ncr1^gfp/gfp^* animals (14). Further studies are necessary to elucidate both the mechanisms driving reduced angiopoietin-2 levels in this model and the contribution of reductions in this cytokine to the systemic vascular phenotype.

Although PH in the *Nfil3^−/−^* and *Ncr1-Gfp* models was accompanied by pulmonary arteriolar muscularization, spontaneous disease in these animals was not associated with significant hypertrophy of the right ventricle. This observation is consistent with the modest degree of PH in these mice, which is substantially less severe than the pulmonary vasoconstriction experienced by mice exposed to chronic hypoxia (10% oxygen), with or without the VEGF receptor inhibitor SU5416 (27). Similar examples of PH in the absence of RV hypertrophy have been reported in other mouse models of PAH, including spontaneous disease in mice bearing targeted mutations in the type II bone morphogenetic protein receptor (*Bmpr2*) (21, 27), as well as in *ApoE^−/−^* mice that develop PH in response to a high-fat Paigen diet (24).

The role of immunity in the pathogenesis of PAH is now well appreciated. Despite this appreciation, understanding of the contribution of specific immune cell subsets to disease remain poorly understood. The current study presents data from two independent genetic models to highlight an important role for NK cells in the regulation of both pulmonary and systemic arterial pressure. When taken together, our findings support an emerging recognition of the capacity of NK cells to regulate the structure and integrity of vascular beds outside the uterus (23, 49) and provide some of the most compelling evidence to date of a role for NK cell impairment in the pathogenesis of PAH. These studies also build on previous work identifying a phenotypic and functional impairment of circulating NK cells in patients with PAH (31) and a potential role for these cells in the pathogenesis and treatment of PAH in rodent models of disease (32, 33). Ongoing and future studies will aim to clarify the precise molecular mechanisms underlying vascular dysfunction in these models of NK cell impairment.

## GRANTS

This work was funded through Canadian Institutes of Health Research Project Grant PJT-152916 and British Heart Foundation Fellowship FS/12/39/29653.

## DISCLOSURES

No conflicts of interest, financial or otherwise, are declared by the authors.

## AUTHOR CONTRIBUTIONS

M.L.O. conceived and designed research; M.T.R., S.D.M., S.J., M.M., M.S., and M.L.O. performed experiments; M.T.R., S.D.M., and M.L.O. analyzed data; M.T.R., S.D.M., and M.L.O. interpreted results of experiments; M.T.R. and M.L.O. prepared figures; M.T.R. and M.L.O. drafted manuscript; M.T.R., H.J.M.B., O.M., N.W.M., F.C., and M.L.O. edited and revised manuscript; M.T.R., S.D.M., S.J., H.J.M.B., O.M., M.S., N.W.M., F.C., and M.L.O. approved final version of manuscript.
